# Two new species of *Myrmedonota* Cameron (Staphylinidae, Aleocharinae) from Mexico

**DOI:** 10.3897/zookeys.464.8549

**Published:** 2014-12-16

**Authors:** Quiyari J. Santiago-Jiménez

**Affiliations:** 1 Museo de Zoología, Facultad de Biología-Xalapa, Universidad Veracruzana, Zona Universitaria, Circuito Gonzalo Aguirre Beltrán s/n, Xalapa, Veracruz, C.P. 91090, MÉXICO

**Keywords:** Lomechusini, false Lomechusini, Nearctic, Neotropical

## Abstract

Two new species of *Myrmedonota* are described from Mexico. Illustrations and a distribution map are provided, as are keys to identify *Myrmedonota* known from the Nearctic and Neotropics. Specimens were collected by means of mercury vapor light traps or flight interception traps.

## Introduction

Recently, Hlaváč et al. (2011) cataloged 207 genera and 2,205 species belonging to the Lomechusini tribe. Although the Lomechusini tribe is a polyphyletic group distributed around the world, a clade of false Lomechusini, distributed exclusively in the Neotropics, has been identified using molecular markers (Elven et al. 2010, 2012, pers. obs.).

The genus *Myrmedonota* was described originally by Cameron in 1920. It only included species distributed in Asia and was recently expanded, when Maruyama et al. (2008) redescribed the genus, to include two new species from North America. Later, Eldredge (2010) described another species from Kansas, U.S.A. The latter author included a key to the species distributed in the America, north of Mexico. Later, Mathis and Eldredge (2014) described two other species of *Myrmedonota* from Mexico and included commentary on the taxonomy and behavior of this genus. Now, I am adding another two species to this genus, one each from the states of Veracruz and Jalisco in Mexico. The specimens match the generic characters outlined by Maruyama et al. (2008) in their redescription.

## Materials and methods

Between 2004 and 2006, on two field trips to Jalisco and Veracruz in Mexico specimens were collected using mercury vapor light traps or flight interception traps. The samples were preserved in 96% ethanol, and some of the specimens were identified as belonging to *Myrmedonota*.

The specimens were observed using a Stemi DV4 stereomicroscope. Photographs from slides were taken using an image processing system (VELAB microscope model VE–633, with Digital LCD model DMS-153). Whereas, habitus photographs were taken using an Stemi 2000-C, with digital camera Canon PowerShot G10. Images were merged using the image stacking software Combine ZP. Illustrations were made based on those photographs of the structures. Permanent microscope slides were prepared using the techniques described by Santiago-Jiménez (2010). The terminology used here follows Santiago-Jiménez (2010), and in some cases Ashe (1984). Holotypes and paratypes were deposited at the Museo de Zoología, Universidad Veracruzana, Xalapa, Veracruz, Mexico. Some paratypes will be deposited in IEXA.

## Taxonomy

### *Myrmedonota* Cameron, 1920

The genus was redescribed by Maruyama et al. (2008). More recently, Eldredge (2010) and Mathis and Eldredge (2014) proposed a diagnosis for the genus, based only partially on the characters used by Maruyama et al. (2008). For example, they didn’t mention nothing about pronotum transverse or medial projection on apodeme that used Maruyama et al. (2008) on their diagnosis. Eldredge (2010) made a key to the species of North America, but it has some problems (e.g. body length in the key does not coincide with body length in the descriptions). To date, the species from Mexico have not been included in any key.

### Taxonomic comments

Mathis and Eldredge (2014) mentioned that pseudo-Lomechusini from the New World belong to Athetini based on a Bayesian analysis run by Elven et al. (2010). However, there is a misunderstanding about the phylogenetic relationships, because the tree was not completely resolved to support the conclusion that *Myrmedonota* belongs to Athetini. However, there is evidence that false (i.e., misidentified) Lomechusini from the Neotropics are a completely different clade from those included in the true Lomechusini and Athetini based on molecular analysis with more taxa and more characters (Santiago-Jiménez and Gusarov, in prep.).

*Diagnosis*. Maruyama et al. (2008) characterized the genus as follows: 1) body surface finely punctate; 2) head with occipital suture; 3) pronotum transverse, >1.5 wider than long; 4) setation on abdomen sparse to moderate; 5) cardo of maxilla covers bases of stipes and lacinia; 6) lacinia extremely narrowed and parallel-sided; 7) mentum almost as long as wide; 8) apodeme of labium with medial projection; 9) 1^st^ segment of labial palpus longer than 2^nd^ segment; 10) each lobe of ligula with 2 setulae.

### Key to *Myrmedonota* species from the Nearctic and the Neotropics

**Table:** 

1	Length of body 3.0 mm or less	**2**
–	Length of body more than 3.0 mm (maximum 4.2 mm)	**6**
2	Pronotum yellowish; spermatheca with proximal end curved over itself (Fig. 20 in Eldredge 2010)	***Myrmedonota heliantha* Eldredge**
–	Pronotum reddish brown, dark brown or black; spermatheca with proximal end not curved over itself	**3**
3	Abdominal segments unicolored, black; spermatheca V–shaped (Fig. 21 in Maruyama et al. 2008)	***Myrmedonota lewisi* Maruyama & Klimaszewski**
–	Abdominal segments bicolored, usually II–IV or only anterior half of IV paler than V–VIII; spermatheca S–shaped, or if V–shaped, then abdominal tergites bicolored, with II–III and base of IV dark brown, and posterior half of IV to VIII black	**4**
4	Abdominal tergites II–IV dark brown, and V–VIII black; spermatheca V–shaped (Fig. 4 in Mathis and Eldredge 2014)	***Myrmedonota shimmerale* Mathis & Eldredge**
–	Abdominal tergites II–IV yellowish to reddish brown, with at most a dark brown spot on each one, and tergites V–VIII darker; spermatheca S–shaped	**5**
5	Abdominal tergites II–IV yellowish with a dark spot on medial area of tergites III–IV (Fig. 2), tergites V–VIII black; apex of median lobe, short, slightly curved ventrally (Fig. 15); spermatheca with apex of the neck plain, as in Fig. 18	***Myrmedonota jaliscensis* sp. n.**
–	Abdominal tergites II–IV reddish brown and V–VIII blackish brown (sometimes medial areas of tergite IV and V blackish brown); apex of median lobe, long, looking more sharply curved ventrally (Fig. 8 in Maruyama et al. 2008); spermatheca with apex of the neck concave (Fig. 12 in Maruyama et al. 2008)	***Myrmedonota aidani* Maruyama & Klimaszewski**
6	Pronotum yellowish to dark brown; elytra bicolored with humeral region yellow and rest of elytra dark brown; abdominal tergites II–IV yellowish and V–VIII dark brown to black (except basal region of tergite V is yellowish); apex of median lobe, slightly curved ventrally (Fig. 6 in Mathis and Eldredge 2014); spermatheca without accessory gland (Fig. 8 in Mathis and Eldredge 2014)	***Myrmedonota xipe* Mathis & Eldredge**
–	Pronotum dark brown to black; elytra not bicolored, humeral region not yellow, elytra entirely brown; abdominal tergites III–V with apical region yellowish brown, appearing paler than the rest (Fig. 1); apex of median lobe, more sharply curved ventrally (Fig. 7); spermatheca with accessory gland close to the neck as in Fig. 10	***Myrmedonota cordobensis* sp. n.**

### Myrmedonota cordobensis sp. n.

Taxon classificationAnimaliaColeopteraStaphylinidae

http://zoobank.org/0DC18D13-34C6-45A4-9978-7BE0C0095556

Figures 1 , 3–10 , 19

#### Type locality.

Mexico, Veracruz: Córdoba, Matlaquiahuitl, 1570m, 18°59'41"N, 96°53'35.1"W, cloud forest, light trap, 6.VII.2006, J. Asiain, J. Márquez, L. Delgado and Q. Santiago leg.

#### Type material.

Holotype male, pinned. Original label: “MÉXICO: Veracruz, Córdoba, Matlaquiahuitl. 6.VII.2006. Bosque Mesófilo de Montaña perturbado, 1,570m, 18°59'41"N, 96°53'35.1"W, ex. trampa de luz. J. Asiain, J. Márquez, L. Delgado y Q. Santiago”/“MUZ-UV-COL-00000065”/”HOLOTYPE *Myrmedonota cordobensis* Santiago-Jiménez, 2014” [red label].

#### Other material.

Paratypes, same data as holotype (42 males, 14 females MUZ-UV, IEXA).

#### Description.

Body length: 3.5–4.1 mm. Most of body black to dark brown; elytra and legs brown; apical region of abdominal segments III–V, usually brown. Pubescence dense to sparse on head, pronotum and elytra, denser on elytra; dorsal surface of abdomen almost glabrous, dense pubescence on ventral surface of abdomen.

Head: Transverse, with or without impression on disc; without protuberance or carinae. Antennal articles 1–3 brown, 4–11 black, tip of 11 brown. Antennal articles 1–2 very elongate, 3–9 elongate, 10 slightly elongate, and 11 very elongate.

Mouthparts: Labrum: with 8 setae on each side of the midline; most of the setae on anterior half; with more than 30 sensory pores on each side of midline; sensillae on apical margin of epipharynx, arranged in a pattern of anterior or α–sensilla, medial or β–sensilla, posterior or γ–sensilla, and lateral or ε–sensilla, one on each side of the midline (see Ashe 1984, Santiago-Jiménez 2010); apico-medial margin of epipharynx not modified to setose or with spinose process; basal region of epipharynx with only four pores, more or less in one transverse row; medial region of epipharynx with more than 50 pores in an irregular array; mesal region of epipharynx without a multiporose sensory structure on each side of the midline; with 8 to 10 pores on mesolateral region. Mandibles: asymmetrical; right mandible with medial tooth on dorsal position; left mandible without tooth; without incisor tooth; with serration on apical half of both mandibles; with large velvety patch wider than half of mandible base, composed of small denticles; prostheca with short setae along entire length, except base, which has a ctenidium; prosthecal setae not bifurcated in medial area. Maxilla: with a row of seven spines and two rows of large setae contiguous with the apical spines on apical third of the lacinia, between two rows of setae there is a glabrous area; the two rows of setae continue with numerous setae on middle third of the lacinia; practically glabrous on the basal third of the lacinia; with pseudopores on the cardo. Labium: with short ligula and divided near base; with a small pair of setulae on each lobe of the ligula (one very short); without medial spines. Prementum with two medial setae, insertions widely separated; medial pseudopore field present; lateral pseudopore field composed of one setose pore, and two asetose pores, with setae on aboral margin of hypoglossa, adoral margin also with setae. Mentum without microsculpture on surface; with scarcely distributed pores on mentum (around 30 pores on each side of the midline), more densely distributed toward the apex.

Thorax: Pronotum transverse, wider on anterior third; surface finely punctured, moderately dense; without reticulate microsculpture; setae moderately dense on surface; with 4 macrosetae along lateral margins, 3 macrosetae on each side of the midline, 2 macrosetae between lateral and medial macrosetae, distributed on anterior half. Scutellum with surface smooth, moderately covered with short setae. Elytra slightly wider on apical area; surface finely punctured, moderately dense; without reticulate microsculpture; setae moderately dense, covering the surface; with 6 macrosetae: 3 on lateral margin, and 3 diagonally placed starting from the base of midline outward. Hind wings well developed, flabellum with 16–17 spines. Mesocoxal acetabula completely margined posteriorly. Mesocoxal cavities moderately separated (approx. 0.20 mm) by meso- and metaventral processes; mesoventral process short (approx. 0.18 mm) with apex truncated; metaventral process medium-sized (approx. 0.56 mm), marginate and with apex acuminate; isthmus distinctly present (approx. 0.09 mm). Legs short, tarsal formula 4–5–5, every leg with an empodium, one seta on empodium and a pair of tarsal claws, each claw with a subbasal tooth.

Abdomen: Subparallel-sided, narrower than elytra, wider around segments IV–V; surface smooth, tergites III–VII almost glabrous, but with a row of 3 macrosetae along posterior margins on each side of midline of every segment and one macroseta closer to the meso-lateral region; tergite VIII (Figs 3–4) with 5 macrosetae on each side of the midline; tergite IX with 4 macrosetae on each side of the midline; tergite X with 4 macrosetae on each side of the midline. Other conspicuous characters are: tergites III–VI with basal impression; sternite IV with a central and transverse reservoir, without glands on basal region, without striae or cuticle vesicles on anterocentral region, without spiracles on basal region, without transversal cuticular impressions on basal region, without pseudopores on basal region.

Secondary sexual structures: Sternite VII of male with external gland on basal region and pseudopores on posterior margin of gland. Tergite VIII of male (Fig. 3) with posterior margin truncate and crenate (around 6–7 denticles), and one lateral protrusion on each external margin. Tergite VII of female without external gland or pseudopores. Tergite VIII of female (Fig. 4) not crenate and without lateral protrusion. Sternite VIII of male and female as illustrated in Figures 5 and 6, respectively.

Aedeagus: Median lobe pear-shaped (Figs 7–8); internal sac of medial lobe with many spinules; median lobe with short, well defined compressor fig; apical lobe curved to the ventral side (visible in lateral view), and pointed; basal ridge convex. Paramere as in Fig. 9; anterodorsal margin of paramerite with prominent sensory pores present beneath the velar sac; hinge zone of paramerite faint, extended from dorsal surface to near articulation between condylite and paramerite; apical process of paramerite clearly articulated anterior to edge of velum; condylite with a line of sensory pores; velum short (less than one half of the length of the paramere). Apical lobe with 4 macrosetae visible (see Eldredge 2010).

Spermatheca: Basal bulb simple, rounded at base; tube S–shaped; internal tube of neck with denticles; with accessory gland (Fig. 10).

**Figure 1.**
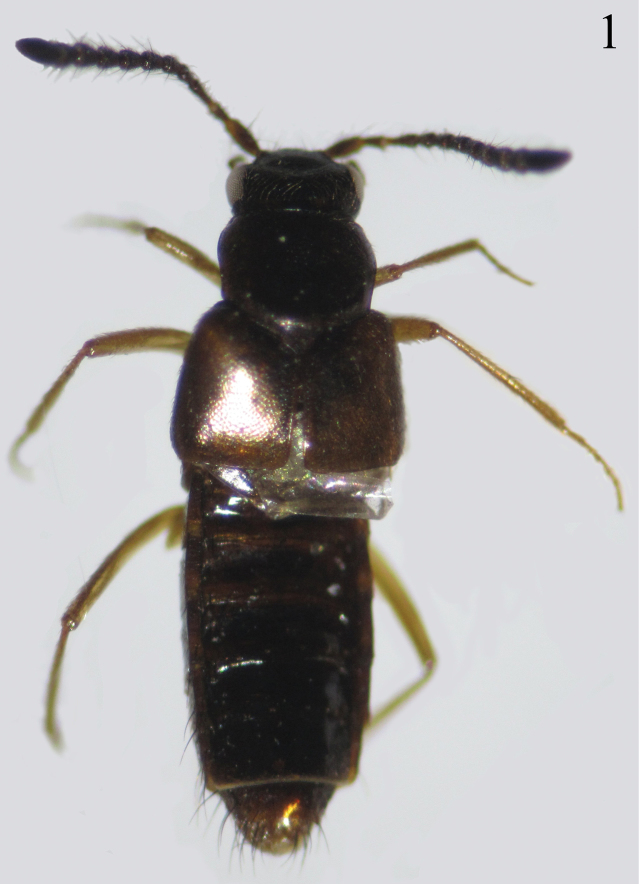
Habitus of *Myrmedonota cordobensis* Santiago-Jiménez, sp. n., male.

#### Remarks.

It is very similar in size to *Myrmedonota xipe*, but *Myrmedonota cordobensis* sp. n. is easy to distinguish because it is darker, the elytra are not bicolored, the apical region of tergites III–V is brown–yellowish, and the spermatheca is different in shape.

#### Etymology.

The name makes reference to the municipality where the specimens were collected, Córdoba in the state of Veracruz.

#### Habitat.

Unknown. The adult specimens were collected with mercury vapor light traps. The larval habitat is not known.

#### Distribution.

*Myrmedonota cordobensis* sp. n. is only known from the type locality in the central region of the state of Veracruz, Mexico. This locality is 1,570 m above sea level, in a disturbed cloud forest. Matlaquiahuitl is the highest mountain in the municipality of Córdoba, Veracruz (Fig. 19).

### Myrmedonota jaliscensis sp. n.

Taxon classificationAnimaliaColeopteraStaphylinidae

http://zoobank.org/BA2F7DF8-089F-4ED8-8CE6-DF555594BBA2

Figures 2 , 11–18 , 19

#### Type locality.

Mexico, Jalisco: Chapala, 4 Km. Ajijic–Chapala, 20°17'48.8"N, 103°12'55.5"W, dry deciduous forest (*Acacia* sp.), flight interception trap, 17.IX.2004, S. Gámez, A. López and Q. Santiago leg.

#### Type material.

Holotype male, pinned. Original label: “MÉXICO: Jalisco, Chapala, 4 Km. Ajijic–Chapala. 15–17.IX.2004. Huizache, 1,620 m, 20°17'48.8"N, 103°12'55.5"W, ex. trampa de intercepción de vuelo. S. Gámez, A. López y Q. Santiago”/“ MUZ-UV-COL-00000603”/”HOLOTYPE *Myrmedonota jaliscensis* Santiago-Jiménez, 2014” [red label].

#### Other material.

Paratypes, same data as holotype (15 males, 5 females MUZ-UV, IEXA).

#### Description.

Body length: 2.6–3.0 mm. Most of body black to dark brown; anterior edge of elytra, abdominal segments III–IV, and legs (except apical half of meso- and metafemur darker) yellowish brown. Densely pubescent on head, pronotum and elytra; dorsal surface of abdomen almost glabrous, densely pubescent on ventral surface of abdomen.

Head: Transverse, with or without impression on disc; without protuberance or carinae. Antennal articles 1–3 brown, 4–11 black, but tip of 11 is brown. Antennomeres 1–3 very elongate, 4–10 elongate, and 11 very elongate.

Mouthparts: Labrum: with 8 setae on each side of the midline; most of the setae on anterior half; with more than 30 (around 32–37) sensory pores on each side of the midline; sensillae on apical margin of epipharynx, arranged in a pattern of anterior or α–sensilla, medial or β–sensilla, posterior or γ–sensilla, and lateral or ε–sensilla, one on each side of the midline (see Ashe 1984, Santiago-Jiménez 2010); apico-medial margin of epipharynx not modified to setose or spinose process; basal region of epipharynx with six pores more or less in one transverse row; medial region of epipharynx with around 30–32 pores in an irregular array; mesal region of epipharynx without a multiporose sensory structure on each side of midline; with several pores (around 8) on mesolateral region. Mandibles: asymmetrical; right mandible with medial tooth on dorsal position; left mandible without tooth; without incisor tooth; with serration between apex and medial area of mandibles; with large velvety patch, wider than half of mandible base, composed of small denticles; prostheca with short hairs along entire length, except base, which has a ctenidium; prosthecal hairs not bifurcated on medial area. Maxilla: with a row of seven spines and two rows of large setae contiguous with the apical spines on apical third of the lacinia, between two rows of setae there is a glabrous area; the two rows of setae continue with numerous setae on middle third of the lacinia; scarcely distributed setae present on basal third of the lacinia; with pseudopores on cardo. Labium: with a short ligula and divided to near the base; with a small pair of setulae on each lobe of the ligula (one very short on the apex); without medial spines. Prementum with two medial setae, insertions widely separated; medial pseudopore field present; lateral pseudopore field composed of one setose pore, and two asetose pores; with setae on aboral margin of hypoglossa, adoral margin with setae too. Mentum without microsculpture on surface; with scarce pores on mentum (around 20–22 pores on each side of midline), more densely toward the apex.

Thorax: Pronotum transverse, wider on anterior third; surface finely punctured, moderately dense; without reticulate microsculpture; setae moderately dense on surface; with 4 macrosetae along lateral margins, 3 macrosetae on each side of the midline, 2 macrosetae between lateral and medial macrosetae distributed on anterior half. Scutellum with reticulate microsculpture, moderately covered with short setae. Elytra slightly wider on apical area; surface finely punctured, moderately dense; without reticulate microsculpture; covered moderately with setae; with 8 macrosetae: 3 on lateral margin, 3 on mesal area, and 2 in diagonal closer to inner border. Hind wings well developed, flabellum with 15 spines (one female had only 10 spines). Mesocoxal acetabula completely margined posteriorly. Mesocoxal cavities moderately separated (approx. 0.16 mm) by meso- and metaventral processes; mesoventral process short (approx. 0.17 mm) with apex truncated; metaventral process medium-sized (approx. 0.56 mm), marginate and with apex acuminate; isthmus distinctly present (approx. 0.07 mm). Legs short, tarsal formula 4–5–5, every leg with an empodium, one seta on empodium and a pair of tarsal claws, each claw with a subbasal tooth.

Abdomen: Subparallel-sided, narrower than elytra, wider around segments IV–V; surface smooth, tergites III–VII almost glabrous, but with a row of 3 macrosetae along posterior margins on each side of the midline of every segment and one macroseta closer to the meso-lateral region; tergite VIII (Figs 11–12) with 5 macrosetae on each side of midline; tergite IX with 4 macrosetae on each side of midline; tergite X with 4 macrosetae on each side of midline. Other conspicuous characters are: tergites III–VI with basal impression; sternite IV with a central and transverse reservoir sac; without glands in basal region; without striae or cuticle vesicles on anterocentral region; without spiracles in basal region; without transversal cuticular impressions in basal region; without pseudopores in basal region.

Secondary sexual structures: sternite VII of male without external gland in basal region. Tergite VIII of male (Fig. 11) with posterior margin truncate and crenate (around 6 denticles), and one lateral protrusion on each side of the midline. Tergite VIII of female (Fig. 12) is not crenate and it has a lateral protrusion. Sternite VIII of male and female as illustrated in Figures 13 and 14, respectively.

Aedeagus: Median lobe pear-shaped (Figs 15–16); with internal sac of median lobe with many spinules; medial lobe with short, well defined compressor fig; apical lobe curved to the ventral side (visible in lateral view), and pointed; basal ridge convex. Paramere as in Fig. 17; anterodorsal margin of paramerite with prominent sensory pores present beneath the velar sac; hinge zone of paramerite faint, extended from dorsal surface to near articulation between condylite and paramerite; apical process of paramerite clearly articulated anterior to edge of velum; condylite with a line of sensory pores; velum short (less than one half of the length of the paramere). Apical lobe with 3 macrosetae visible.

Spermatheca: Basal bulb simple, rounded at base; tube S–shaped; internal tube of neck with denticles; without accessory gland (Fig. 18).

**Figure 2.**
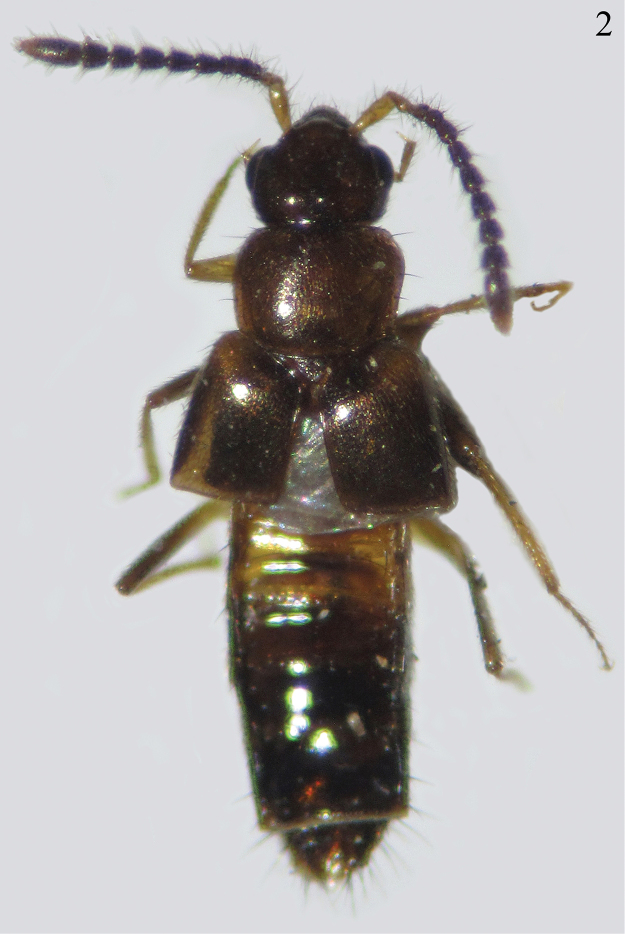
Habitus of *Myrmedonota jaliscensis* Santiago-Jiménez, sp. n., male.

**Figures 3–10.**
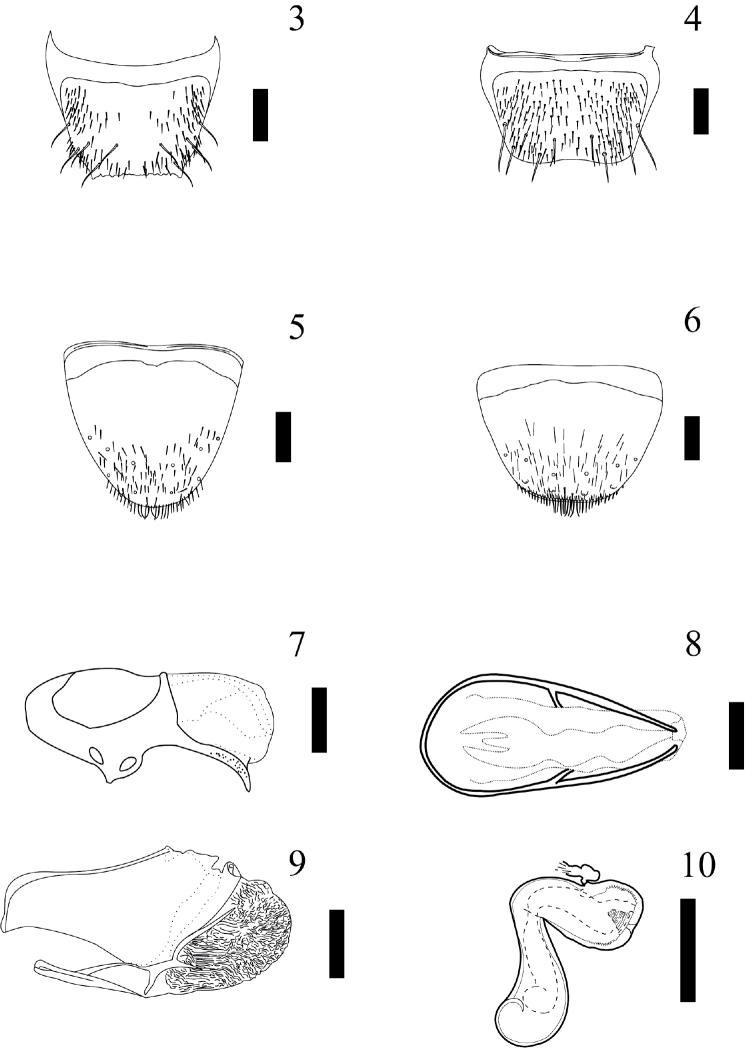
*Myrmedonota cordobensis* Santiago-Jiménez, sp. n. male (**3, 5, 7–9**) and female (**4, 6, 10**). **3** tergite VIII **4** tergite VIII **5** sternite VIII (note that macrosetae were lost, only pores were illustrated) **6** sternite VIII (note that macrosetae were lost, only pores were illustrated) **7** median lobe, lateral view **8** median lobe, dorsal view **9** paramere, outer lateral view **10** spermatheca. Scale bar = 0.2 mm, except scale bar of spermatheca = 0.1 mm.

**Figures 11–18.**
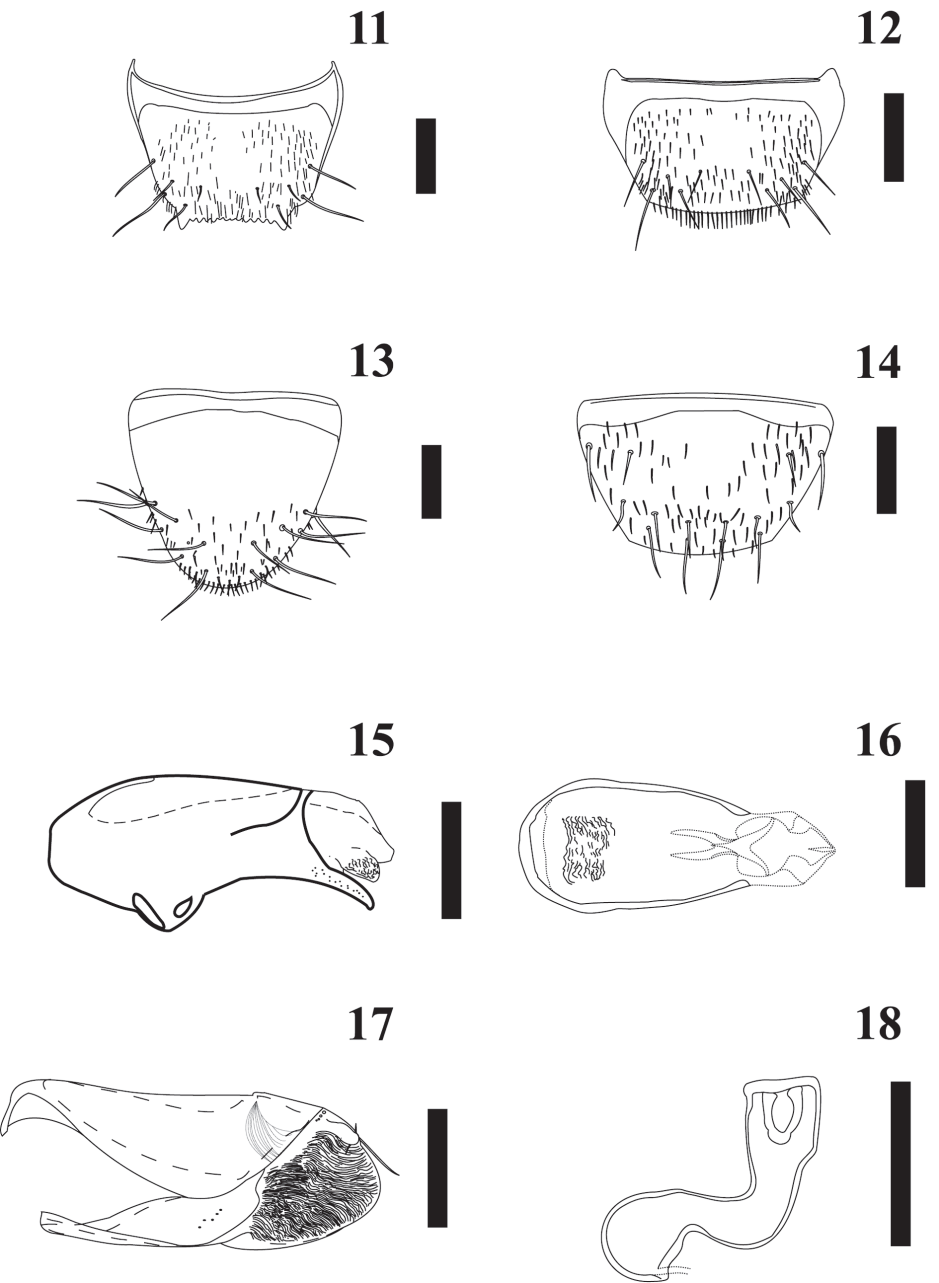
*Myrmedonota jaliscensis* Santiago-Jiménez, sp. n. male (**11, 13, 15–17**) and female (**12, 14, 18**). **11** tergite VIII **12** tergite VIII **13** sternite VIII **14** sternite VIII **15** median lobe, lateral view **16** median lobe, dorsal view **17** paramere, outer lateral view **18** spermatheca. Scale bar = 0.2 mm, except scale bar of spermatheca = 0.1 mm.

#### Remarks.

*Myrmedonota jaliscensis* is 3 mm or less in size and is easy to distinguish from other species: from *Myrmedonota heliantha* because the proximal end of the spermatheca is not curved over itself; from *Myrmedonota lewisi* because the abdomen is bicolored; from *Myrmedonota shimmerale* because the spermatheca is S–shaped; and finally, from *Myrmedonota aidani* because tergites II–IV are yellowish with a dark spot on medial area of tergites III–IV, and the differently shaped spermatheca.

#### Etymology.

The name makes reference to the state of Jalisco, Mexico, where the specimens were collected.

#### Habitat.

Unknown. The adult specimens were collected with interception flight traps. The larval habitat is not known.

#### Distribution.

**Figure 19.**
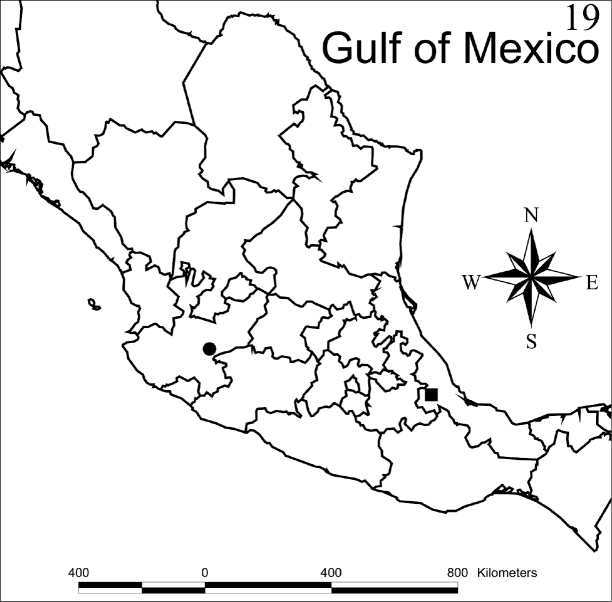
Collection sites of *Myrmedonota cordobensis* Santiago-Jiménez, sp. n. (black square) and *Myrmedonota jaliscensis* Santiago-Jiménez, sp. n. (black circle).

*Myrmedonota jaliscensis* sp. n. is only known from the type locality around Lake Chapala in Jalisco state, Mexico (Fig. 19). This locality is 1,620 m above sea level, where it is common to find *Acacia* sp. trees, the common name of which is Huizache.

## Discussion

More species of *Myrmedonota* are being described from the Nearctic and Neotropical regions, and here I have described two new species, and it is possible that more species will be discovered in the future. Although Eldredge (2010) and Mathis and Eldredge (2014) presented a new diagnosis of *Myrmedonota*, it is not clear what specimens they used to select their diagnostic characters. Specimens reviewed here matched with diagnostic characters proposed by Eldredge (2010) and Mathis and Eldredge (2014); however, as mentioned above, they didn’t mention nothing about pronotum transverse or medial projection on apodeme that used Maruyama et al. (2008) on their diagnosis. Moreover, there is an inconsistency about labial palpomeres from diagnosis by Mathis and Eldredge (2014) compared to previous diagnosis by Eldredge (2010). I think it should be labial palpomeres I and III subequal in length, not II and III as mentioned by Mathis and Eldredge (2014). Therefore, I suggest we follow the redescription proposed by Maruyama et al. (2008) because they reviewed the type species of *Myrmedonota* and it has been useful to diagnose Nearctic and Neotropical species. Diagnostic characters should be proposed in a future analysis by mean of synapomorphies on a phylogenetic context.

Misunderstandings in Elven et al. (2010) about the limits of the Lomechusini-Athetini complex are causing confusion for people working with both tribes. That phylogeny was not completely resolved, and the main conclusion is that the species of false Lomechusini from the Neotropics belong to a different clade, but it was not possible to conclude whether they should be part of Athetini.

Finally, it is quite interesting that more species of *Myrmedonota* are being described from the Neotropics because new biogeographical questions are also emerging. Future efforts should aim to test whether *Myrmedonota* is a monophyletic clade that includes Oriental, Nearctic and Neotropical species, and to investigate the relationships between species.

## Supplementary Material

XML Treatment for Myrmedonota cordobensis

XML Treatment for Myrmedonota jaliscensis
